# 晚期非小细胞肺癌治疗策略的演变：从2009放眼2012

**DOI:** 10.3779/j.issn.1009-3419.2010.03.16

**Published:** 2010-03-20

**Authors:** David R. GANDARA, Philip C. MACK, Tianhong LI, Primo N. LARA, JR, Roy S. HERBST, 永波 杨

**Affiliations:** 1 Division of Hematology and Oncology, University of California Davis Cancer Center, Sacramento, CA; 2 Department of Oncology, Montefiore-Einstein Cancer Center, Bronx, NY; 3 Department of Thoracic, Head and Neck Medical Oncology, University of Texas M. D. Anderson Cancer Center, Houston, TX; 4 天津医科大学总医院，天津市肺癌研究所，天津市肺癌转移与肿瘤微环境重点实验室

新近的临床试验资料，包括2009年美国临床肿瘤协会(ASCO)年会上报道的资料，有力地推动了对晚期非小细胞肺癌(NSCLC)患者的现代治疗策略的改革。此改革蕴含的主题包括：(1)基于组织学类型的治疗，(2)预测性生物标记物，以及(3)维持治疗。尽管这些新信息对制定治疗决策过程的作用很可能随着时间的推移而演变，但治疗模式的改革已日益凸显。在此，我们将展望这一演进的远景，从2009放眼2012.

迄今为止，晚期NSCLC及大多数癌症的治疗策略仍主要以经验为依据。近年来，正如2007年ASCO指南中所述，对于身体状况良好的患者，可推荐采用基于铂类的双药方案作为一线化疗，即顺铂或卡铂联合另一种药(经验性地在包括吉西他滨、多西紫杉醇、紫杉醇、培美曲塞和长春瑞滨的这一组药物中选择) ^[1]^。最近，对于适合使用贝伐单抗的患者(主要是非鳞癌类型和之前没有咳血的患者)，这一血管表皮生长因子(VEGF)靶向药物联合化疗可作为一种选择^[2]^。另外，刚刚公布的FLEX(First-Line Erbitux in Lung Cancer)试验显示了西妥昔单抗联合化疗的获益，尽管这一表皮生长因子受体(EGFR)靶向单克隆抗体尚未被批准用于NSCLC^[3]^。在使用4-6个周期之后，基于铂类的化疗一般即会被终止，而且患者治疗结束，并采取“观察和等待”的策略。直到现在，这一治疗策略唯一值得注意的例外是那些采用贝伐单抗作为一线化疗的患者，贝伐单抗通常可作为单药继续使用直至疾病进展。

2009年，在治疗领域又出现了新的治疗选择(如表 1所示)。推荐治疗策略将各种特征联合起来，这些特征可归类为临床(患者身体状况)特征、组织学类型特征和分子生物学特征。首先，基于肿瘤组织学类型为NSCLC患者选择化疗方案成为一种合理的选择，至少对于化疗药物培美曲塞是这样。三项随机Ⅲ期试验显示培美曲塞对于非鳞状组织学类型的NSCLC最有效，这使得FDA严格限制了该药物的使用^[4-6]^。到目前为止，尚无确凿证据支持其它NSCLC常用化疗药物可基于组织学类型来制定策略。例如，西南肿瘤学组(SWOG)数据库的分析显示基于组织学类型为患者选用抗微管药物紫杉烷类化合物或者长春花类的有效性无差别，而且是否吉西他滨更适合鳞癌类型仍然存在争议^[7, 8]^。

其次，尽管近年来研究报道了许多与化疗和靶向治疗相关的潜在的预测性标志物，但至今无一被证实可“改变实践”。然而，NSCLC中为研究目的而进行的生物标志物检测和临床实践之间的壁垒已经被攻破。基于IPASS(Iressa Pan-Asian Study)试验最新公布数据，现在可以明确地得出这样的结论：对于新近被诊断为晚期NSCLC且已知伴有EGFR活化的酪氨酸激酶功能域突变的患者，一线治疗采用EGFR酪氨酸激酶抑制剂(TKI)优于化疗^[9]^。在IPASS中，一项完全在东亚进行的Ⅲ期临床试验，所纳入的患者均为腺癌，主要为从不吸烟者和女性患者，晚期NSCLC患者被随机分为采用EGFR-TKI吉非替尼或紫杉醇/卡铂化疗作为一线治疗。总体上，无病进展生存期(PFS)在吉非替尼组占优，考虑到研究人群该结果在意料之中。然而，令人惊奇的是，在本试验中，EGFR突变状态预示着患者的预后结果。存在EGFR活化突变的约60%的患者中，吉非替尼治疗组的PFS显著改善。相反，存在野生型EGFR的患者中，化疗组的PFS占优。值得注意的是，两治疗组的总生存期无差别。然而，这些资料为新推荐的治疗策略的子策略提供了依据(图 1所示)，新推荐的治疗策略为：突变阳性的患者采用EGFR-TKI。尽管近年来报道了许多其它预测性生物标志物，但目前仅EGFR突变状态被认为可改变治疗方案。

**1:**
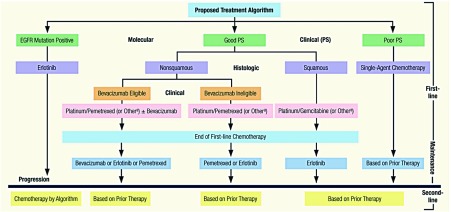
晚期非小细胞肺癌的推荐治疗策略：2009 Proposed Treatment Algorithm for Advanced-Stage Non–Small-Cell Lung Cancer: 2009

图 1中显示的治疗方案演变的最后一步与一线化疗完成后的维持治疗有关。尽管之前的临床试验探讨过这一观点，但对于它的采纳并无有力的资料来支持。取而代之的是，采取所谓的“药物休整期”并联合观察和等待，已成为常规方案。然而，现在已有两项大型Ⅲ期试验(JMEN和SATURN)表明对于一线化疗完成后达到疾病缓解或者疾病稳定的NSCLC患者，分别采用培美曲塞或者厄洛替尼作为维持治疗是切实可行的选择^[6, 10]^。必须强调的是，鉴于某种程度上上述两项试验中曾接受该研究药物的安慰剂组中患者人数有限(< 20%)，在阐述其结果时应该加以注意。另外，两项试验中术语“维持”本身是不恰当的，因为培美曲塞或者厄罗替尼均非起始一线治疗的组成部分，因此也并非随后的“维持”治疗。相反的是，培美曲塞或者厄洛替尼均为临床进展出现之前采用的新药。因此，就概念而言，术语“巩固”或者“早期二线治疗”可能更为准确。无论如何，在2009年，所谓的维持治疗是一个值得考虑的新的治疗选择。但并非每一例晚期NSCLC患者均可从此方案中获益，其最适合某些亚组的患者。例如，早期采用额外治疗是一线化疗达到疾病缓解或疾病稳定但仍存在相应癌症症状的患者的较佳选择。

如果2009治疗策略确实可行，那我们未来前进的方向在哪里？图 2呈现了晚期NSCLC的假定治疗策略，其有望在2012年变为现实。与2009治疗策略相比，生物学特征成为2010推荐治疗策略的基础。尽管我们已经认识到目前不断发展的分子生物学在未来数年可能使我们走向无法预知的方向，但目前存在数个个体化生物标志物，及更多的广泛地以基因组策略为基础的生物标记物，这将为基于分子的治疗策略的制定打下基础^[11]^。简便起见，图 2列出了一个基于有前景的个体化预测性分子生物标志物(ERCC1，RRM1，TS)的治疗策略，每一个标志物均不同程度地被多项研究的临床试验数据所支持，而且目前可以从CLIA(Clinical Laboratory Improvement Amendments)授权的实验室获取这些数据^[12-17]^。

**2:**
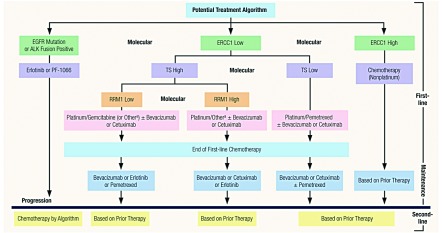
晚期非小细胞肺癌的潜在治疗策略（患者身体状况良好）：2012 Potential Treatment Algorithm for Advanced-Stage Non-Small-Cell Lung Cancer (Good Performance Status): 2012

在2012推荐治疗策略中，为应用EGFR TKI的靶向治疗进行分子筛选仍然是第一步。同样，在该靶向治疗的分类中，基于有前景的近期报道，我们推测最近公布的ALK抑制剂PE-1066，及其它在研的类似药物，将会被加入到异常检测呈阳性的NSCLC患者的治疗策略中^[18]^。虽然对于为化疗而进行的现有生物标志物的检测何时可跨越基础实验与治疗标准的基线还存在着争议，但我们认为很可能在2012年临床肿瘤学者们将以分子生物学作为基础来制定治疗策略^[19]^。许多临床试验采用了不同的研究方法，但是都关注了通过分子选择而进行的个体化治疗，包括BATTLE项目(Biomarker-integrated Approaches of Targeted Therapy for Lung Cancer Elimination)、SWOG0720和癌症与白血病B组，目前这些研究均在进行中^[20-22]^。这些研究及其它研究被认为是从以分子为基础的方案向治疗计划过渡的现代“试验场”，其中新的成功方案可转化应用与临床实践。我们认为这些进展将使肿瘤领域关于现代医疗的所有讨论者受益。总而言之，为癌症治疗鉴别预测性生物标志物，包括新的和昂贵的药物，理应减少药物使用量，使处方仅限于最有可能获益的患者：一种成本-效益策略。
